# Clinical pharmacists’ interventions for preventing adverse events in critically ill neonates in Qatar: an economic impact analysis

**DOI:** 10.1080/20523211.2023.2291508

**Published:** 2024-01-02

**Authors:** Ola Yakti, Daoud Al-Badriyeh, Mohammed Rijims, Mohammed Abdelaal, Omar Alsoukhni, Moza Al Hail, Palli Valapila Abdulrouf, Wessam El-Kassem, Fouad Abounahia, Rasha Kaddoura, Dina Abushanab

**Affiliations:** a Pharmacy Department, Hamad General Hospital, Hamad Medical Corporation, Doha, Qatar; b College of Pharmacy, QU Health, Qatar University, Doha, Qatar; c Pharmacy Department, Hamad Bin Khalifa Medical City, Hamad Medical Corporation, Doha, Qatar; d Pharmacy and Drug Control, Ministry of Public Health, Doha, Qatar; e Pharmacy Department, Al Jalila Children's Specialty Hospital, Dubai, United Arab Emirates; f Neonatal Intensive Care Unit, Hamad Medical Corporation, Doha, Qatar; g Pharmacy Department, Heart Hospital, Hamad Medical Corporation, Doha, Qatar

**Keywords:** Adverse drug event, Neonates, cost savings, economics, intervention, pharmacist

## Abstract

**Objective:**

This study aimed to assess the overall economic impact of clinical pharmacist interventions in the neonatal ICU (NICU) in Qatar.

**Methods:**

A retrospective review of neonates’ records was performed over a 3-month duration in the NICU of Qatar to determine the total economic benefit of clinical pharmacist interventions. The total benefit of interventions was calculated by considering the cost avoidance due to preventable adverse drug events (ADEs) and the cost savings associated with the revised resource use due to interventions. Sensitivity analyses were conducted to ensure the robustness and generalizability of the results.

**Results:**

A total of 513 interventions were analyzed, involving 150 neonates. Most of the drug-related problems were related to therapy dosing, followed by drug choice appropriateness, the addition of prophylactic treatment, and administration frequency. The overall annual benefit was estimated at QAR 4,178,352 (1,147,584), which consisted of cost avoidance of QAR 1,050,680 (USD 288,648) and an overall cost saving of QAR −6091 (USD −1673).

**Conclusions:**

While the clinical pharmacist interventions led to increased resource utilisation and associated costs, when considering the avoided costs of ADEs, the overall clinical pharmacist practices in the NICU setting were economically beneficial.

## Introduction

Healthcare-related costs are rising globally. Many of these costs are attributed to medication use and medication errors (Aspden & Aspden, 2007). Approximately 1.5 million preventable adverse events are reported annually in the United States, incurring a cost of approximately 3.5 billion dollars (Aspden & Aspden, 2007). Later studies suggest that the United States spends about 40 billion dollars per year on patients affected by medication errors and more than 21 billion dollars per year on preventable medication errors (Abushanab et al., 2021; Arredondo et al., 2021). One study found that around 45% of the adverse events were preventable and reported that although medication errors have ranged, to mention a few, from pharmaceutical labelling, route-specific problems due to challenges with medication formulation design, accessibility, and human error (Arredondo et al., 2021), the most serious adverse events happened during ordering the medication or while administering it. In the same study, it was reported that the majority of these adverse events were near misses and slips, rather than stemming from a lack of knowledge (Arredondo et al., 2021). The situation is similar in the intensive care unit (ICU) settings as a systematic review found that the frequencies of medication errors ranged widely, with rates spanning from 8.1 to 2344 occurrences per 1000 patientdays (Wilmer et al., 2010). Similarly, Adverse drug events (ADEs) exhibited variability, ranging from 5.1 to –87.5 instances per 1000 patientdays (Wilmer et al., 2010).

This is considered more important as neonatal intensive care stays are among the most expensive types of hospitalisation (Congress, 1987).

Several studies have shown the critical role of the pharmacist in reducing medication errors, shortening the duration of stay, and minimising the overall cost in different healthcare settings, including the ICU (Arredondo et al., 2021; Houso et al., 2022; Menezes et al., 2020). Studies have been conducted in Qatar to assess the economic impact of pharmacists’ interventions in a general tertiary hospital (Abushanab, Atchan, et al., 2023), cardiology (Al-Badriyeh et al., 2023), and cancer care settings (Abushanab, Gulied, et al., 2023). These studies found that pharmacists’ interventions were associated with a positive annual total benefit, including a positive cost avoidance. Although these clinical pharmacist interventions were associated with an increased cost of resource use, this increased cost was overtaken by the cost of avoidance generated. This role has contributed to this as the clinical pharmacist has been involved in several aspects of patient care, from evaluating the efficacy and safety of the administered medication, medication reconciliation, prevention of medication errors, and medical residents’ education (Arredondo et al., 2021). There is a lack of data about the economic impact of clinical pharmacist interventions against drug-related problems (DRPs) in neonatal ICUs in Qatar. The availability of such data will assist decision-makers and policymakers in better judging the need for clinical pharmacy services, including the justification of salaries for personnel.

The study aims to evaluate the economic benefit of clinical pharmacist interventions in preventing adverse drug events (ADEs) in the neonatal intensive care unit (NICU) of Hamad Medical Corporation (HMC) in Qatar.

## Materials and Methods

### Study setting

The study was conducted at the NICU in HMC. HMC is the main provider of healthcare in Qatar, which incorporates 13 hospitals and is considered one of the leading corporations providing healthcare services in the Middle East region (Abushanab et al., 2021). The HMC tertiary NICU setting includes a total of 120 beds and is the largest and most specialised facility in the country that provides specialised services for neonates who are often admitted within the first 24 h after birth for a variety of reasons, including premature birth, difficulties during delivery, and/or signs of problems during the first few days of life (Abushanab et al., 2021; Heyman et al., 2003).

### Study design

The study was a retrospective review of clinical pharmacist interventions, defined as any action a pharmacist took that altered the neonate course of therapy (Dooley et al., 2004). Clinical interventions included in this study were acquired from the clinical intervention forms available in the Cerner medical database. When pertinent information could not be obtained from the clinical intervention sheet, information was directly acquired from the neonate’s Cerner medical record.

### Study population

The clinical pharmacist interventions examined in this study were related to those performed on neonates admitted to the NICU. The study sample consisted of interventions carried out on neonates during a 3-month follow-up period, encompassing the months of March 2018, 15 July to 15 August 2018, and January 2019.

### Ethics approval

The study was approved by the Medical Research Center, HMC (MRC-01-19-110).

***Inclusion criteria:***

- All clinical pharmacist interventions on neonates admitted to the NICU throughout the specified follow-up durations (in March 2018, from 15 July to 15 August 2018, and in January 2019).
- Clinical pharmacist interventions were for neonates who continuously took at least one medication during their hospitalisation, either for a new indication or for a previous one.
- Interventions recommended by a clinical pharmacist.
- Interventions recommended by a clinical pharmacist and approved by a physician and, hence, implemented.

***Exclusion criteria:***

- Interventions carried out by a staff/operational pharmacist (non-clinical), without the clinical pharmacist looking after the neonate.
- Interventions that were rejected by the clinicians. Regardless of the intervention and the evidence behind it, if this is not accepted by the clinician, this will not be implemented.

### Economic evaluation

The main objective of this study was to assess the economic impact of clinical pharmacist interventions to prevent ADEs.

#### Cost savings

The total cost savings from clinical pharmacist interventions were determined by subtracting the cost of after-clinical pharmacy intervention therapy from the cost of before-clinical pharmacist intervention therapy when this is in positive values. Here, the cost of after intervention was based on the original therapy duration until intervention, plus the cost of therapy after the change, based on the duration of its full course. While the cost before intervention was based on the duration of therapy before intervention.

The following equation was used to calculate the cost savings:

$$\begin{array}{rl} Cost\;savings & =cost\;of\;therapy\;resource\;use\;before\;intervention\;minus\;cost\\ & \;of\;therapy\;resource\;use\;after\;intervention,where\;positive\;cost\;saving\\ & \;represents\;reduced\;cost\;with\;intervention,while\;negative\;cost\;saving\\ & \;represents\;added\;cost\;with\;intervention. \end{array}$$

#### Cost avoidance

Cost avoidance refers to the cost that was prevented by eliminating the occurrence of ADEs through clinical pharmacist interventions (Nesbit et al., 2001). The likelihood of an ADE in the absence of the intervention was determined using the method described by Nesbit et al., which categorised the likelihood as 0 (none), 0.01 (very low), 0.1 (low), 0.4 (medium), or 0.6 (high) (Chen et al., 2017).

The cost of an ADE was estimated based on a conservative assumption that it would result in an additional two days of hospital stay in the corresponding unit, in accordance with relevant literature studies (Abushanab, Atchan, et al., 2023; Abushanab, Gulied, et al., 2023; Al-Badriyeh et al., 2023; Chen et al., 2017).

In this study, a 3-month prescription refills cost was used for chronic disease medications, while for acute diseases, we used the duration according to the prescription order or the national HMC guideline.

The total cost avoidance was calculated by multiplying the average of the total probabilities of all ADEs by the total cost of ADEs as shown in the equation below:

$$\begin{array}{rl} Cost\;avoidance & =average\;probability\;of\;avoided\;ADE\;with\;the\;interventions\\ & \;multiplied\;by\;the\;cost\;of\;ADE. \end{array}$$

#### Total benefit analysis

The cumulative benefit of the clinical pharmacist interventions was assessed on a 3-month and a projected annual basis. The monetary value of the interventions’ benefit was determined by calculating the sum of the cost savings and cost avoidance achieved through the interventions.

The following equation was used to calculate the total economic impact:

Total economic impact=cost savings+cost avoidance due to interventions

### Expert panel

Following the approach suggested by Nesbit et al., an expert panel consisting of three clinical pharmacists, each with more than 10 years of clinical experience in the field and one neonatologist, was convened to determine the probabilities of ADEs in the absence of interventions (Nesbit et al., 2001). Each panel member estimated the likelihood of an ADE occurring without an intervention. The average probability of an ADE in the absence of each intervention was then considered. The neonatologist validated the generated probability estimates of ADEs.

Appendix 1 depicts an overall diagram of study.

### Perspective

The study was conducted from the perspective of the public NICU in Qatar. Of note, in the Qatari public healthcare system, all medications and non-medication resources are provided free of cost to hospitalised patients (i.e. Qatari citizens and residents).

### Cost inputs

To determine the monetary value of resources, the costs associated with medications, non-medication-based resources (such as laboratory and diagnostic tests), and hospital stay were obtained from the pharmacy, finance, and costing departments at HMC (‘Qatar Inflation Rate', 2021). All costs were adjusted to the financial year 2023 using the Qatari Health Consumer Price Index. The monetary values were presented in Qatari Riyal (QAR) and the USD. No discounting was applied because outcomes were not projected beyond a 1-year time horizon.

### Sample size

This study is not a comparative study, where the sample size used in similar literature studies varies based on factors such as the size of the setting and the prevalence of underlying conditions (Walsh et al., 2017). Local studies in Qatar (Abushanab, Atchan, et al., 2023; Abushanab, Gulied, et al., 2023; Al-Badriyeh et al., 2023), as well as international studies (Gallagher et al., 2014; Malani et al., 2013; Sebaaly et al.), in secondary/tertiary care settings have reported sample sizes ranging from <100 to <2000, but predominantly <500. Considering the anticipated incidence of interventions in the study setting, a preliminary investigation indicated that a 3-month duration would be sufficient to include over 500 interventions for analysis.

In line with our previous studies (Abushanab, Atchan, et al., 2023; Abushanab, Gulied, et al., 2023; Al-Badriyeh et al., 2023), to account for any potential variations in pharmacist vigilance during the institutional review process of pharmacists’ performance, the sample size included the first month after the annual staff performance evaluation in the NICU, the last month of the year before the evaluation, and a middle month of the year.

### Statistical analysis

Data were tabulated for each neonate and analyzed using the IBM SPSS (Statistical Package for the Social Sciences) version-24. The data were presented as numerical and percentage measures for categorical variables and as mean and standard deviation measures for continuous variables. Kruskal–Wallis and Chi-Square tests were used to detect any significant differences among the three follow-up months, i.e. March 2018, July/August 2018, and January 2019.

### Sensitivity analysis

Sensitivity analyses evaluated the uncertainty surrounding main cost and probability inputs. A one-way sensitivity analysis (OWSA), targeting one uncertain input variable at a time, was performed to assign a ±20% variation range of the base case value of the cost of the ADEs, using a triangular type of random value distribution. A probabilistic sensitivity analysis (PSA), targeting several probabilistic inputs at once, was used to assign an uncertainty range of ±15% of the base case values of the probabilities of ADEs, using a triangular-type distribution, and based on 10,000 simulations. All analyses were performed via Monte Carlo simulation, using @Risk-5.7 (Palisade Corporation, NY). All outcomes were presented as probability curves.

## Results

### Characteristics of neonates and interventions

A total of 150 neonates in the NICU were included in this study, with a total of 513 clinical interventions during the follow-up period of this study. Of the study population, 63 (42.0%) were females and 87 (58.0%) were males. The mean age for both sexes was 3.62 ± 0.51 days. There were no significant differences in baseline characteristics between the three follow-up months. Further details regarding the baseline characteristics of the included population are summarised in Table 1.

**Table 1.** Neonates’ demographics among the study periods.

Variable average (±standard deviation)/frequency (%)	Total number of neonates (*n* = 150)	March 2018 (*n* = 47)	July–August 2018 (*n* = 37)	January 2019 (*n* = 66)	*p* value
Sex					
Male	78 (52.0%)	19 (41.7%)	18 (48.6%)	41 (62.1%)	0.09
Female	72 (4.0%)	28 (58.3%)	19 (51.4%)	25 (37.9%)	
Age	3.62 ± 0.51	4.0 ± 0.20	4.0 ± 0.0	3.11 ± 0.31	0.40
Weight	2.65 ± 1.05	3.0 ± 1.2	2.25 ± 0.78	2.59 ± 1.0	0.32
Nationality					
Arab	100 (66.6%)	32 (68.8%)	25 (67.6%)	43 (65.2%)	0.11
Asian (non-Arab)	39 (26.0%)	11 (22.9%)	9 (24.3%)	19 (28.8%)	
African (non-Arab)	4 (2.7%)	1 (2.1%)	1 (2.7%)	2 (3.0%)	
Others	7 (4.7%)	3 (6.2%)	2 (5.4%)	2 (3.0%)	
Ward type					
Inpatient (critical)	149 (99.3%)	47 (100%)	37 (100%)	65 (98.5%)	0.18
Inpatient (non-critical)	1 (0.7%)	0 (0%)	0 (0%)	1 (1.5%)	

The most common intervention intercepted by the clinical pharmacists was related to the medications’ dosing (63.2%), followed by interventions related to drug choice appropriateness (26.8%), addition of prophylactic treatment (3.7%), and administration frequency (1.9%). In Table 2, a description of the categories of the interventions is presented with examples, together with the associated average probability of avoided ADEs as per category.

**Table 2.** Summary of the probability of avoided adverse drug events and categories of the clinical pharmacist interventions.

Probability of avoided adverse drug event	Numbers and categories of the interventions
0.005	3 interventions; ‘Increase in medication dose’, ‘addition of another medication’, ‘change in medication route’.
0.01	131 interventions; ‘Discontinuation of a medication’, ‘addition of another medication’, ‘change in medication route’, ‘increase in medication frequency’, ‘increase in medication dose’, ‘decrease in medication duration’, ‘decrease in medication frequency’, ‘decrease in medication dose’, ‘addition of a prophylactic agent’.
0.055	39 interventions; ‘Discontinuation of a medication’, ‘addition of another medication’, ‘increase in medication dose’, ‘increase in medication frequency’, ‘increase in medication duration’, ‘decrease in medication dose’.
0.1	232 interventions; ‘Discontinuation of a medication’, ‘addition of another medication’, ‘switching to alternative medication’; ‘increase in medication dose’, ‘increase in medication frequency’, ‘addition of a prophylactic agent’, ‘decrease in medication dose’.
0.205	1 intervention; ‘Decrease in medication dose’.
0.25	19 interventions; ‘Discontinuation of a medication’, ‘decrease in medication dose’, ‘increase in medication dose’
0.4	88 interventions; ‘Discontinuation of a medication’, ‘decrease in medication dose’, ‘addition of lab test’,
0.6	1 intervention; ‘Switching to alternative medication’

### Economic analysis

#### Cost savings

The overall added cost associated with the interventions was QAR 822,741 (USD 226,028), while the overall reduced cost before the interventions was QAR 816,650 (USD 224,293). Therefore, the overall cost saving, due to the pharmacist-led interventions, was in negative, i.e. QAR −6091 (USD −1673). The added and reduced costs with interventions as per different intervention type categories can be seen in Table 3.

**Table 3.** Added resource cost, reduced resource cost, and cost of avoidance according to clinical pharmacist intervention types.

Type of interventions	Overall added cost with interventions, QAR (USD)	Overall reduced cost with interventions, QAR (USD)	Overall cost avoidance, QAR (USD)
Addition of another medication	56,471 (15,514)	0 (0)	57,560 (15,813)
Discontinuation of a medication	0 (0)	102,071 (28,034)	110,433 (30,339)
Switching to alternative medication	426 (117)	1253 (344)	20,306 (5579)
Addition of a prophylactic agent during hospitalisation	2262 (621)	0 (0)	5779 (1588)
Change in medication route	0 (0)	11 (3)	1250 (343)
Change in medication strength	3 (1)	9 (2)	2187 (601)
Change in medication dose	761,140 (209,104)	710,462 (195,128)	810,834 (222,757)
Change in medication duration	718 (197)	1093 (300)	12,887 (3540)
Change in medication frequency	1662 (457)	1751 (481)	10,700 (2940)
Addition of a lab test	60 (16)	0 (0)	18,744 (5149)
Total	822,742 (226,028)	816,650 (224,293)	1,050,680 (288,648)

QAR: Qatari Riyal, USD: United States Dollar.

#### Cost avoidance

The overall cost avoidance due to the interventions over 3 months was QAR 1,050,679 (USD 288,648). Table 3 summarises cost avoidance associated with each intervention type category.

#### Total benefit analysis

Overall economic benefits in favour of the clinical pharmacist interventions are summarised in Table 4. Considering the sum of cost savings and cost avoidance, the total economic benefit over 3 months was calculated to be QAR 1,044,588 (USD 286,896). In addition, the average total benefit was QAR 6964 (USD 1913) per neonate and QAR 2036 (USD 559) per intervention.

**Table 4.** Outcomes of total benefit analysis.

Outcome	Value, QAR (USD)
Overall added cost after interventions per 3 months	822,741 (226,028)
Overall reduced cost before therapy interventions per 3 months	816,650 (224,293)
Overall cost saving per 3 months	−6091 (−1673)
Overall cost avoidance per 3 months	1,050,680 (288,648)
Total benefit per 3 months	1,044,588 (286,896)
Projected total benefit per 1 year	4,178,352 (1,147,584)

QAR: Qatari Riyal, USD: United States Dollar.

### Sensitivity analysis

**Figure 1.**
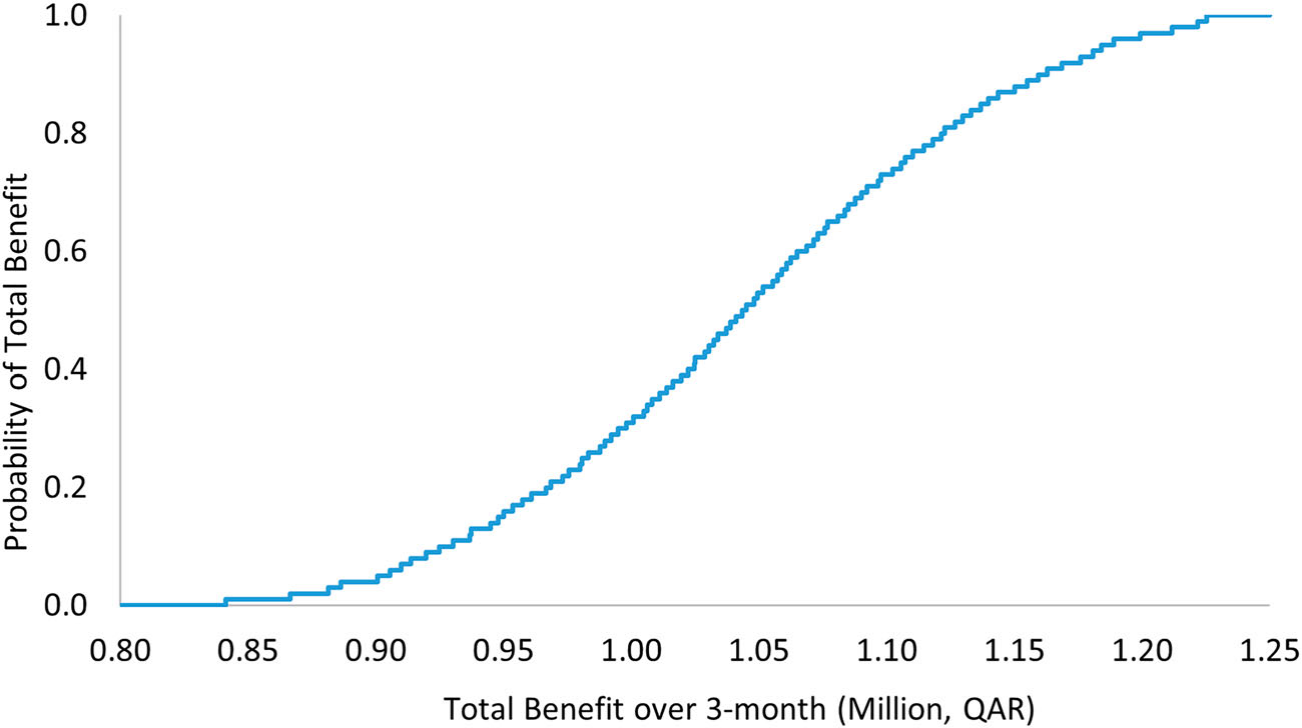
Total benefit probability curve over a 3-month period (one-way sensitivity analysis).

**Figure 2.**
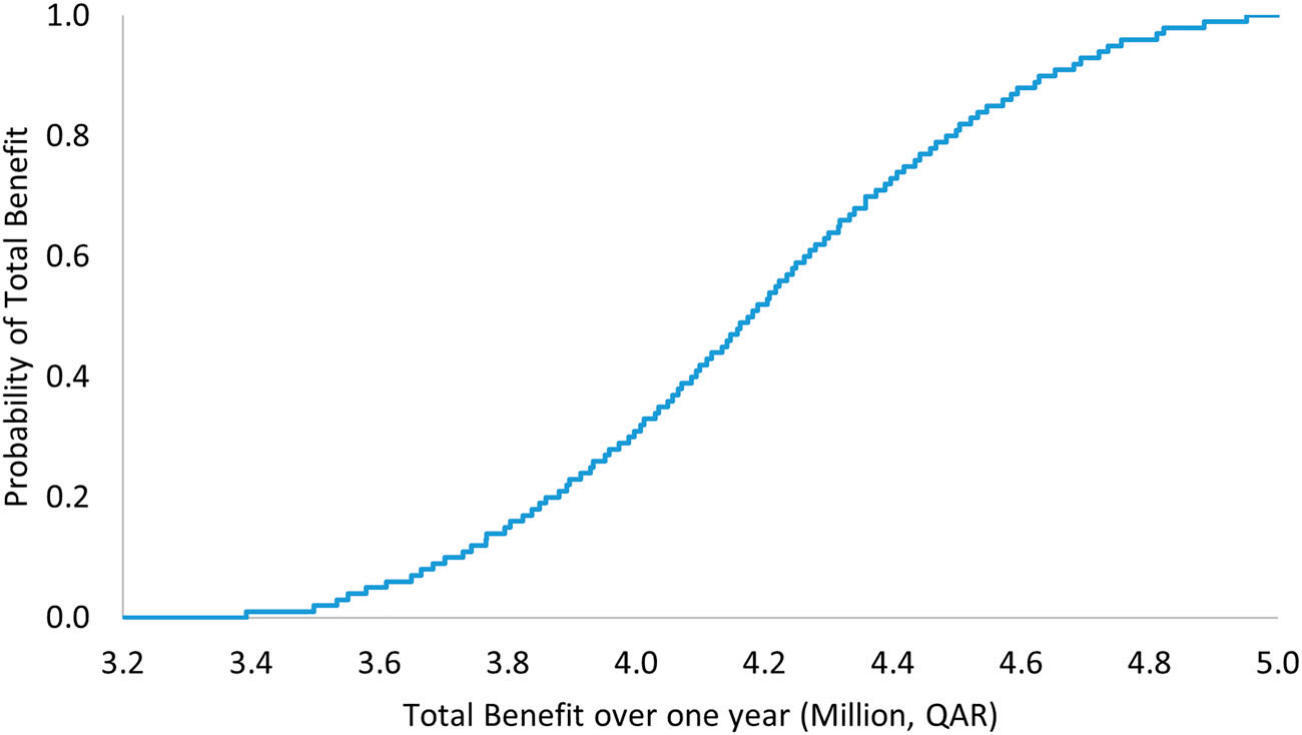
Total benefit probability curve over a 1-year period (one-way sensitivity analysis).

**Figure 3.**
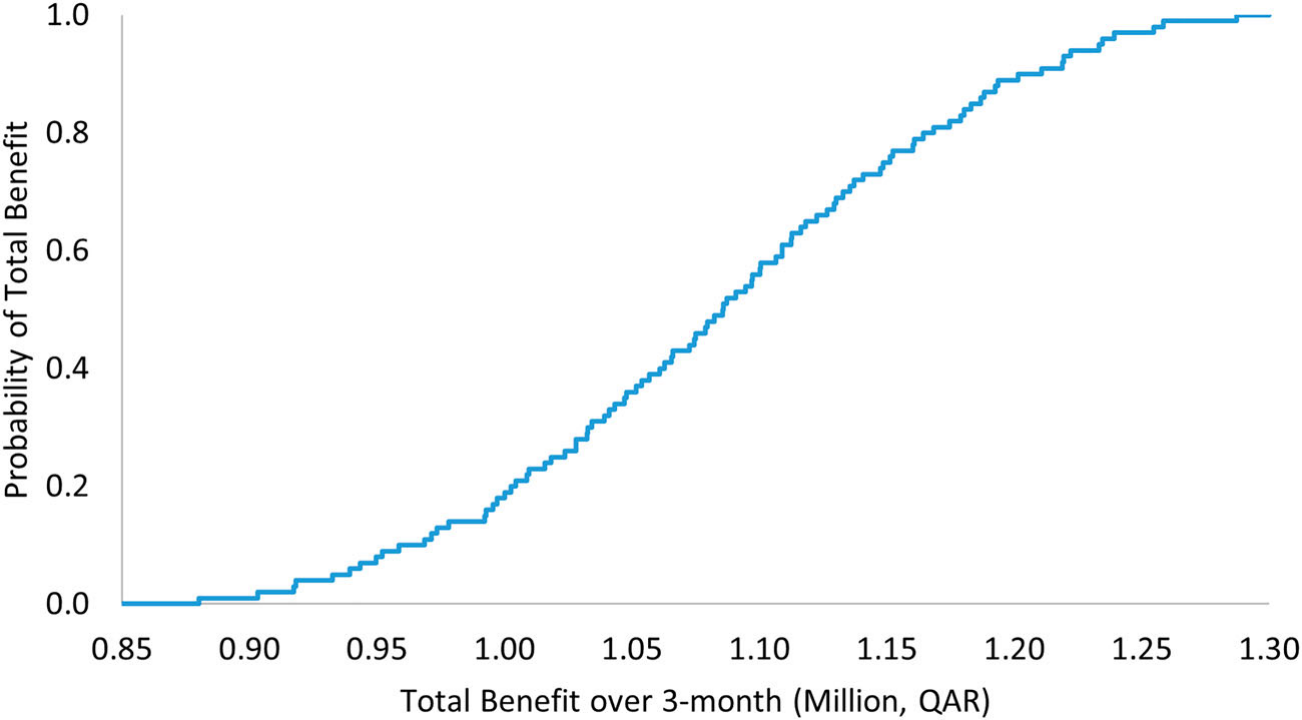
Total benefit probability curve over a 3-month period (probabilistic sensitivity analysis).

**Figure 4.**
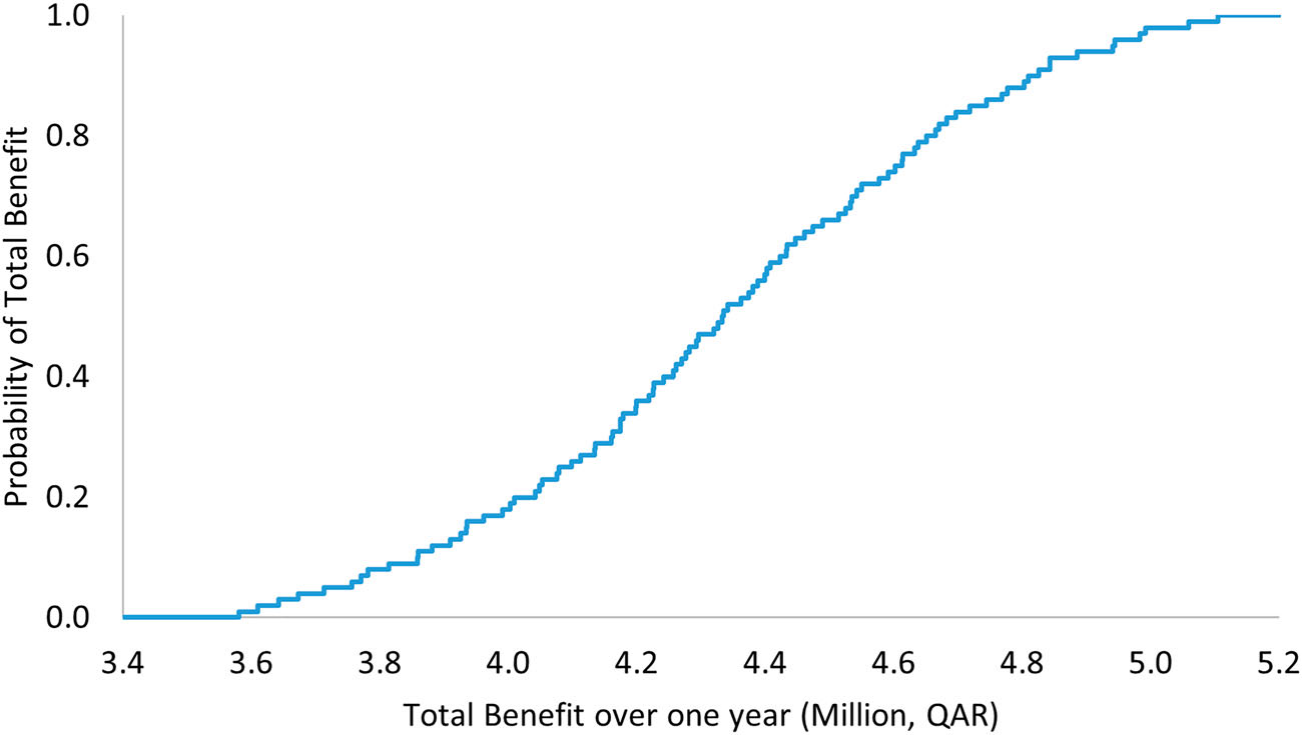
Total benefit probability curve over a 1-year period (probabilistic sensitivity analysis).

The results of OWSA demonstrated robustness against the uncertainty in the cost of the ADE, as reported in Table 5 and Figures 1 and 2. The PSA also showed that there is a 100% probability that the pharmacist-led intervention is associated with positive total benefit with a mean of QAR 1,084,895 (297,966), 95% CI 880,264–1,287,332 (241,764–353,565) over 3 months (Table 6 and Figure 3), and with a mean of 4,339,020 (1,191,711), 95% CI 3,581,233–5,104,675 (983,585–1,401,998) annually (Table 6 and Figure 4).

**Table 5.** Outcomes of one-way sensitivity analysis with their uncertainty distributions.

Variable	Point estimate, QAR (USD)	Variation range	Projected total benefit per 1-year range, QAR (USD)	Total benefit per 3-month, QAR (USD)
Cost of adverse drug event	7810 (2146)	Triangular distribution, QAR 6248, 7810, 9372 (USD 1716, 2146, 2575)	Mean: 4,178,410 (1,147,600), 95% CI 3,392,196 to 4,951,184 (931,666 to 1,359,842)	Mean: 1,044,278 (286,811), 95% CI 841,334 to 1,225,348 (231,072 to 336,542)

**Table 6.** Outcomes of probabilistic sensitivity analysis with their uncertainty distributions.

Variable	Point estimate, QAR (USD)	Variation range	Projected total benefit per 1-year range, QAR (USD)	Total benefit per 3-month, QAR (USD)
Very low probability for ADE	0.01	Triangular distribution, 0.009, 0.01, 0.012	Mean: 4,339,020 (1,191,711), 95% CI 3,581,233 to 5,104,675 (983,585 to 1,401,998)	Mean: 1,084,895 (297,966), 95% CI 880,264 to 1,287,332 (241,764 to 353,565)
Low probability for ADE	0.1	Triangular distribution, 0.09, 0.1, 0.12		
Low to moderate probability for ADE	0.2	Triangular distribution, 0.17, 0.2, 0.23		
Low to moderate probability for ADE	0.3	Triangular distribution, 0.26, 0.3, 0.35		
Moderate probability for ADE	0.4	Triangular distribution, 0.34, 0.4, 0.46		
Moderate to high probability for ADE	0.5	Triangular distribution, 0.43, 0.5, 0.58		
High probability for ADE	0.6	Triangular distribution, 0.51, 0.6, 0.69		

QAR: Qatari Riyal, USD: United States Dollar, CI: confidence interval.

**Figure 5.**
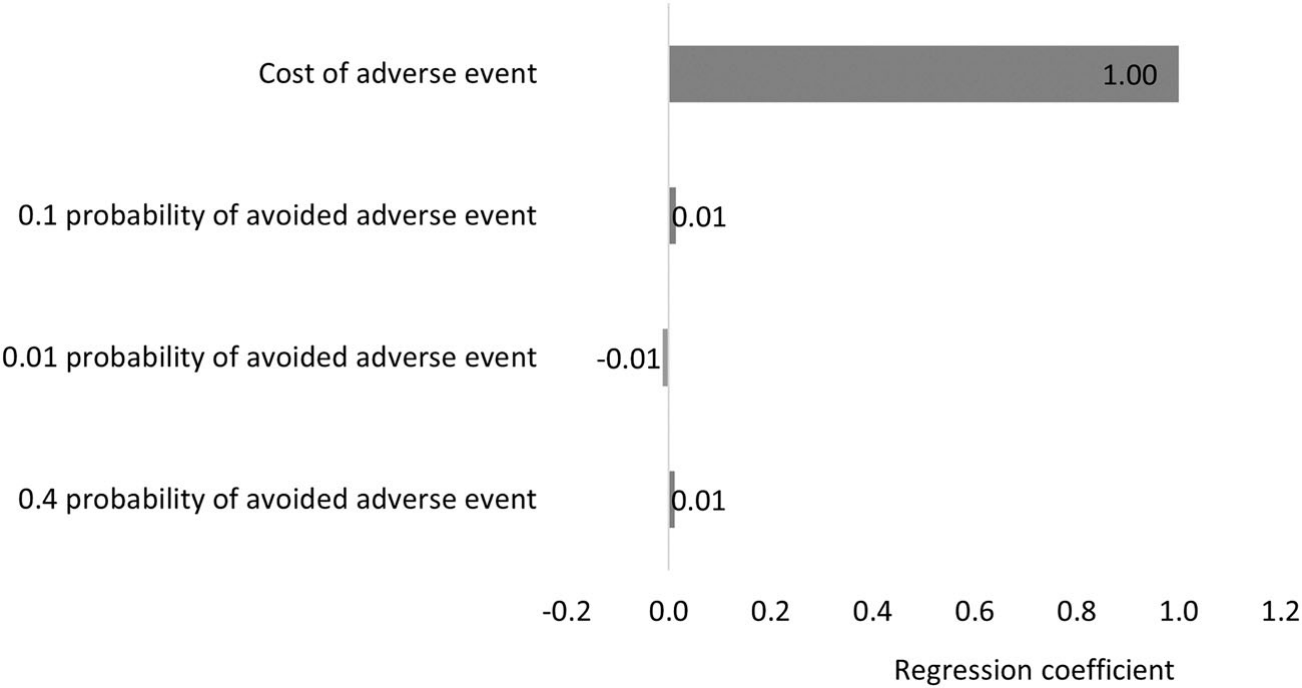
A regression tornado diagram of elements and their effect on the outcome.

A regression Tornado analysis demonstrated that the main contributor to the outcome was the cost of ADE, followed by 0.1 and 0.01 probabilities of avoided ADE (Figure 5). Table 5 shows the results of sensitivity analyses with their uncertainty distributions.

## Discussion

This is the first study to demonstrate the economic impact of clinical pharmacist interventions in the NICU setting in Qatar. A study conducted in a general tertiary hospital in Qatar by our group found that clinical pharmacists’ interventions led to a total of USD 621,106 annual benefit, including a positive cost avoidance of USD 203,260 (Abushanab, Atchan, et al., 2023). Similarly, a study conducted in cardiology settings in Qatar found that pharmacists’ interventions led to cost savings and cost avoidance of USD −3169 and USD 441,616, respectively, yielding a total benefit of USD 1,753,789 per year (Al-Badriyeh et al., 2023).

The results of the current study showed that the clinical pharmacist interventions reduced the cost of resource consumption, leading to cost savings and cost of ADEs avoidance, with a yearly overall monetary value of QAR 4,178,352 (USD 1,147,584).

The interventions that added to the cost of resource use the most were the addition of other medications and the switch to alternative medication. The cost avoidance that drove the overall economic benefit was primarily based on the addition of medications, discontinuation of medications, and addition of prophylactic agents.

Only a few studies have evaluated the economic value of the clinical interventions in the ICU settings. Menezes et al. (2020) have estimated the economic impact of clinical pharmacists in pediatric intensive care units (PICU). In their study, savings were measured over 1-year duration based on (i) clinical pharmacist interventions from prescription checking, (ii) individualised doses of four antibiotics, and (iii) comparison of drug dispensing systems before and after the decentralisation of pharmacy services. In the study, a total of 73 clinical pharmacist interventions were conducted, out of which 13 enabled the assessment of their economic impact, resulting in a yearly saving of USD 633.38. The personalised dosing of four antibiotics resulted in cost savings of USD 8754.46 per year, and the decentralisation of pharmacy services led to annual savings of USD 28,770.52. Here, unlike our study, the cost of ADEs was not the key driver of the study outcome. In another study, by Kim et al. (2019), the authors evaluated the economic impact of pharmacist interventions in the NICU. The study showed that, over 6 months, the total cost avoidance was USD 135,419.30, and the total cost saving was USD 57.78. Unlike our study, pharmacists, not clinical pharmacists, were involved in the Kim et al study. Moreover, the economic evaluation was conducted by analyzing clinical interventions from prescription reviews, total parenteral nutrition consults, and clinical pharmacokinetic consultation service reports delivered by pharmacists in a tertiary hospital, and not by analyzing the cost of ADEs as the case in our current study. Despite these differences, and consistent with our results, noting the lesser extent of impact, the pharmacist interventions led to a positive cost avoidance and a positive cost saving. The observed variation in economic impact between the studies could be attributed to variances in clinical practice approaches and the cost structure of medical services.

Despite that our findings provide valuable insights into the overall economic impact of clinical pharmacist interventions, supported by sensitivity analyses, it is crucial to interpret these results within the context of the specific setting and sample characteristics. The neonatal population, the nature of drug-related problems identified, and the nature of health care system and resource use may differ in other NICU settings.

The current study has several limitations. The nature of the retrospective research design, which has the inherent disadvantage of bias, may cause the overall benefit to be overestimated or underestimated. This is especially true given that daily reporting of clinical interventions is advised but not mandatory and not necessarily documented in a timely manner. Moreover, for the cost of avoided ADEs, it is impossible to follow up neonates until ADEs that did not take place as results of interventions, and then have these accurately estimated, differently with different interventions. Thus, it was assumed that the cost of ADEs would be the same for all ADEs, leading to an additional two days stay in the hospital. This assumption is consistent with previous literature studies, where sensitivity analyses accounted for the uncertainty (Abushanab, Atchan, et al., 2023; Abushanab, Gulied, et al., 2023; Al-Badriyeh et al., 2023; Chen et al., 2017). In addition, a 3-month follow-up period in our study may not accurately reflect the actual economic gains throughout the whole year. Based on the available information, however, there is no compelling rationale to anticipate any significant deviations in observations based on other months, particularly given how the 3-month follow-up was chosen as a sample size, in addition to the lack of significant differences in neonates’ characteristics between the 3 months of the follow-up duration. Finally, generalizability-wise, despite that our findings provide valuable insights into the overall economic impact of clinical pharmacist interventions, the results of this analysis are specific to the Qatari setting and should not be extrapolated to neonates in different settings given the variations in the drug-related problems identified, nature of health care systems, and resource utilisation.

In conclusion, the clinical pharmacists’ interventions toward the rational use and management of medications in the NICU at HMC resulted in a positive overall economic benefit. The result strongly supports the notion that extending the role of clinical pharmacists in the NICU, and possibly other medical specialties would deliver a noteworthy economic advantage to healthcare systems.

## References

[CIT0001] Abushanab, D., Atchan, M., Elajez, R., Elshafei, M., Abdelbari, A., Al Hail, M., Abdulrouf, P. V., El-Kassem, W., Ademi, Z., Fadul, A., Abdalla, E., Diab, M. I., & Al-Badriyeh, D. (2023). Economic impact of clinical pharmacist interventions in a general tertiary hospital in Qatar. *PLoS One*, *18*(6), e0286419. 10.1371/journal.pone.0286419 37262042 PMC10234553

[CIT0002] Abushanab, D., Gulied, A., Hamad, A., Abu-Tineh, M., Abdul Rouf, P. V., Al Hail, M., El-Kassem, W., El Hajj, M. S., & Al-Badriyeh, D. (2023). Cost savings and cost avoidance with the inpatient clinical pharmacist interventions in a tertiary cancer care hospital. *Journal of Oncology Pharmacy Practice*, *29*. 10.1177/10781552231160275 36946146

[CIT0003] Abushanab, D., Rouf, P. A., Al Hail, M., Kamal, R., Viswanathan, B., Parappil, H., Elkassem, W., Al-Shaibi, S., & Al-Badriyeh, D. J. C. T. (2021). Cost-effectiveness of oral versus intravenous Ibuprofen therapy in preterm infants with patent ductus arteriosus in the neonatal intensive care setting: A cohort-based study. *Clinical Therapeutics*, *43*(2), 336–348. e337. 33431169 10.1016/j.clinthera.2020.12.004

[CIT0004] Al-Badriyeh, D., Kaddoura, R., AlMaraghi, F., Homosy, A., Hail, M. A., El-Kassem, W., Rouf, P. V. A., Fadul, A., Mahfouz, A., Alyafei, S. A., & Abushanab, D. (2023). Impact of clinical pharmacist interventions on economic outcomes in a cardiology setting in Qatar. *Current Problems in Cardiology*, *48*(9), 101838. 10.1016/j.cpcardiol.2023.101838 37244514

[CIT0005] Arredondo, E., Udeani, G., Horseman, M., Hintze, T. D., & Surani, S. (2021). Role of clinical pharmacists in intensive care units. *Cureus*, *13*(9), e17929. 10.7759/cureus.17929 34660121 PMC8513498

[CIT0006] Aspden, P., & Aspden, P. (2007). *Preventing medication errors*. National Acad. Press.

[CIT0007] Chen, C.-C., Hsiao, F.-Y., Shen, L.-J., & Wu, C.-C. J. M. (2017). The cost-saving effect and prevention of medication errors by clinical pharmacist intervention in a nephrology unit. *Medicine*, *96*(34). 10.1097/MD.0000000000007883 PMC5572025 28834903

[CIT0008] Congress, U. J. H. t. c. s. (1987). Office of Technology Assessment Neonatal intensive care for low-birth-weight infants: Cost and effectiveness. *JAMA*, *38*.

[CIT0009] Dooley, M. J., Allen, K. M., Doecke, C. J., Galbraith, K. J., Taylor, G. R., Bright, J., & Carey, D. L. J. B. j. o. c. p. (2004). A prospective multicentre study of pharmacist initiated changes to drug therapy and patient management in acute care government funded hospitals. *British Journal of Clinical Pharmacology* *57*(4), 513–521. 15025751 10.1046/j.1365-2125.2003.02029.x PMC1884463

[CIT0010] Gallagher, J., Byrne, S., Woods, N., Lynch, D., & McCarthy, S. (2014). Cost-outcome description of clinical pharmacist interventions in a university teaching hospital. *BMC Health Services Research*, *14*(1), 177. 10.1186/1472-6963-14-177 24742158 PMC4020601

[CIT0011] Heyman, E., Morag, I., Batash, D., Keidar, R., Baram, S., & Berkovitch, M. J. P. (2003). Closure of patent ductus arteriosus with oral ibuprofen suspension in premature newborns: A pilot study. *Pediatrics*, *112*(5), e354. 14595076 10.1542/peds.112.5.e354

[CIT0012] Houso, A., Hamdan, M., & Falana, H. (2022). Cost benefit analysis of clinical pharmacist interventions in medical intensive care unit in Palestine medical complex: Prospective interventional study. *Saudi Pharmaceutical Journal*, *30*(12), 1718–1724. 10.1016/j.jsps.2022.09.017 36601501 PMC9805959

[CIT0013] Kim, Y., Rho, J., Suh, Y., Choi, K., Lee, E., Lee, E., & Choi, C. (2019). 4CPS-234 Pharmacist interventions in neonatal intensive care unit and associated cost avoidance and cost savings. *European Journal of Hospital Pharmacy*, *26*(Suppl 1), A178–A178. 10.1136/ejhpharm-2019-eahpconf.383%J

[CIT0014] Malani, A. N., Richards, P. G., Kapila, S., Otto, M. H., Czerwinski, J., & Singal, B. (2013). Clinical and economic outcomes from a community hospital's antimicrobial stewardship program. *American Journal of Infection Control*, *41*(2), 145–148. 10.1016/j.ajic.2012.02.021 22579261

[CIT0015] Menezes, B. d. M., Lazaretto, F. Z., Lima, L. H., Schwambach, K. H., & Blatt, C. R. J. R. d. C. F. B. e. A. (2020). Economic impact of the clinical pharmacist interventions in the pediatric intensive care unit. *Journal of Basic and Applied Pharmaceutical Sciences*, *41*, 1–8.

[CIT0016] Nesbit, T. W., Shermock, K. M., Bobek, M. B., Capozzi, D. L., Flores, P. A., Leonard, M. C., Long, J. K., Militello, M. A., White, D. A., & Barone, L. D. J. A. j. o. h.-s. p. (2001). Implementation and pharmacoeconomic analysis of a clinical staff pharmacist practice model. *American Society of Health-System Pharmacists*, *58*(9), 784–790. 10.1093/ajhp/58.9.784 11351918

[CIT0017] Qatar Inflation Rate. (2021). https://tradingeconomics.com/qatar/inflation-cpi

[CIT0018] Sebaaly, J., Parsons, L. B., Pilch, N. A., Bullington, W., Hayes, G. L., & Easterling, H. Clinical and financial impact of pharmacist involvement in discharge medication reconciliation at an Academic Medical Center: A prospective pilot study. (0018-5787 (Print)). 10.1310/hpj5006-505 PMC4568111 26405342

[CIT0019] Walsh, E. K., Hansen, C. R., Sahm, L. J., Kearney, P. M., Doherty, E., Bradley, C. P. J. P., & Safety, d. (2017). Economic impact of medication error: A systematic review. *Pharmacoepidemiology and Drug Safety*, *26*(5), 481–497. 28295821 10.1002/pds.4188

[CIT0020] Wilmer, A., Louie, K., Dodek, P., Wong, H., & Ayas, N. (2010). Incidence of medication errors and adverse drug events in the ICU: A systematic review. *BMJ Quality & Safety*, *19*, e7. 10.1136/qshc.2008.030783 20671079

